# PTH[1-34] improves the effects of core decompression in early-stage steroid-associated osteonecrosis model by enhancing bone repair and revascularization

**DOI:** 10.1371/journal.pone.0178781

**Published:** 2017-05-31

**Authors:** Chen-he Zhou, Jia-hong Meng, Chen-chen Zhao, Chen-yi Ye, Han-xiao Zhu, Bin Hu, Boon Chin Heng, Yue Shen, Tiao Lin, Xiao-bo Yang, Zhong-li Shi, Wei-liang Shen, Shi-gui Yan

**Affiliations:** 1 Department of Orthopedic Surgery, Second Affiliated Hospital, School of Medicine, Zhejiang University, Hangzhou, China; 2 Orthopedics Research Institute of Zhejiang University, Hangzhou, China; 3 Faculty of Dentistry, Department of Endodontology, The University of Hong Kong, Pokfulam, Hong Kong; 4 Department of Orthopaedic Surgery, First Affiliated Hospital of Sun Yat-sen University, Guangzhou, China; Nanjing Medical University, CHINA

## Abstract

Steroid-associated osteonecrosis (SAON) might induce bone collapse and subsequently lead to joint arthroplasty. Core decompression (CD) is regarded as an effective therapy for early-stage SAON, but the prognosis is unsatisfactory due to incomplete bone repair. Parathyroid hormone[1–34] (PTH[1–34]) has demonstrated positive efficacy in promoting bone formation. We therefore evaluated the effects of PTH on improving the effects of CD in Early-Stage SAON. Distal femoral CD was performed two weeks after osteonecrosis induction or vehicle injection, with ten of the ON-induced rabbits being subjected to six-week PTH[1–34] treatment and the others, including ON-induced and non-induced rabbits, being treated with vehicle. MRI confirmed that intermittent PTH administration improved SAON after CD therapy. Micro-CT showed increased bone formation within the tunnel. Bone repair was enhanced with decreased empty osteocyte lacunae and necrosis foci area, resulting in enhanced peak load and stiffness of the tunnel. Additionally, PTH enlarged the mean diameter of vessels in the marrow and increased the number of vessels within the tunnels, as well as elevated the expression of BMP-2, RUNX2, IGF-1, bFGF and VEGF, together with serum OCN and VEGF levels. Therefore, PTH[1–34] enhances the efficacy of CD on osteogenesis and neovascularization, thus promoting bone and blood vessels repair in the SAON model.

## Introduction

Steroid-associated osteonecrosis (SAON), the most common form of non-traumatic ON, often occurs following steroid treatment for many non-orthopaedic medical conditions [1–3], with a morbidity rate of 9% to 40% [4]. Patients with SAON often require total hip arthroplasty (THA) after collapse of femoral heads, which accounts for more than 10% of THA performed. Moreover, some patients, especially those with younger age, need revision surgery due to dislocation, infection or osteolysis. Therefore, an effective treatment modality is needed for patients with early-stage SAON to slow OA progression.

Core decompression (CD) is one of the least invasive surgical procedures for early-stage osteonecrosis, aimed at removing necrotic bone, and facilitating bone healing, revascularization of subchondral bone and preventing subsequent joint collapse[5, 6]. CD could also reduce intraosseous pressure, decrease venous congestion and improve capillary blood flow [7, 8]. However, the prognosis is rather poor because of incomplete bone reconstruction and weakening of the trabecular bone within and around the necrotic region, which could lead to failure in preventing progressive collapse [9, 10]. Many adjunctive therapies with CD surgery have been attempted for SAON [11–13], but none of these resulted in any significant improvement.

Parathyroid hormone (PTH) exerts a potent anabolic effect on bone through the PTH receptor (PTH1R) [14, 15]. Recombinant human PTH [1–34] has been approved by the US food and drug administration (FDA) for treating osteoporosis in postmenopausal women and men who have high risk of bone fracture. Intermittent administration of PTH greatly stimulates bone formation, leading to a net gain in bone mass and/or strength [16, 17]. Meanwhile, another study found that PTH could stimulate the release of vascular endothelial growth factor (VEGF) in vitro [18], which is regarded as an essential mediator of angiogenesis.

Based on these known clinical effects of PTH [1–34], we hypothesize that the combination of CD with PTH [1–34] administration will enhance bone repair and revascularization, thereby improving the prognosis of SAON. To test this hypothesis, this study will establish an SAON model to investigate (1) the effects of PTH on improving of the efficacy of CD on SAON; (2) the effects of PTH on the newly formed bone and biomechanical properties of the bone tunnel, as well as osteogenesis around the tunnel and beneath the articular cartilage; (3) the effects of PTH on neovascularization within the tunnel and in the bone marrow; (4) the effects of PTH on the expression of local and systemic osteogenic and angiogenic markers.

## Materials and methods

### Animals

The experimental protocol was approved by the Institutional Animal Care and Use Committee of the Second Affiliated Hospital, School of Medicine, Zhejiang University (Register ID No.: 2015–391; Date: 7 May 2015). The whole experiment was performed according to the Guide for the Care and Use of Laboratory promulgated by the United States National Institutes of Health. Animals were held in a facility under a temperature range from 18°C to 25°C and regular day/night cycle, being allowed free access to food and water. Rabbits were monitored daily to evaluate the signs of pain, distress, or moribundity visually and their weights were measured three times a week. Animals, exhibiting the signs above or 10% acute weight reduction, were to be humanely euthanized prior the experimental endpoint. All animals were euthanized by an overdose of pentobarbital (90 mg/kg, Sigma Chemical Co., St. Louis, MO, USA) at the endpoint. All efforts were made to minimize suffering during the whole experiment.

### SAON model establishment

**Fig 1:**
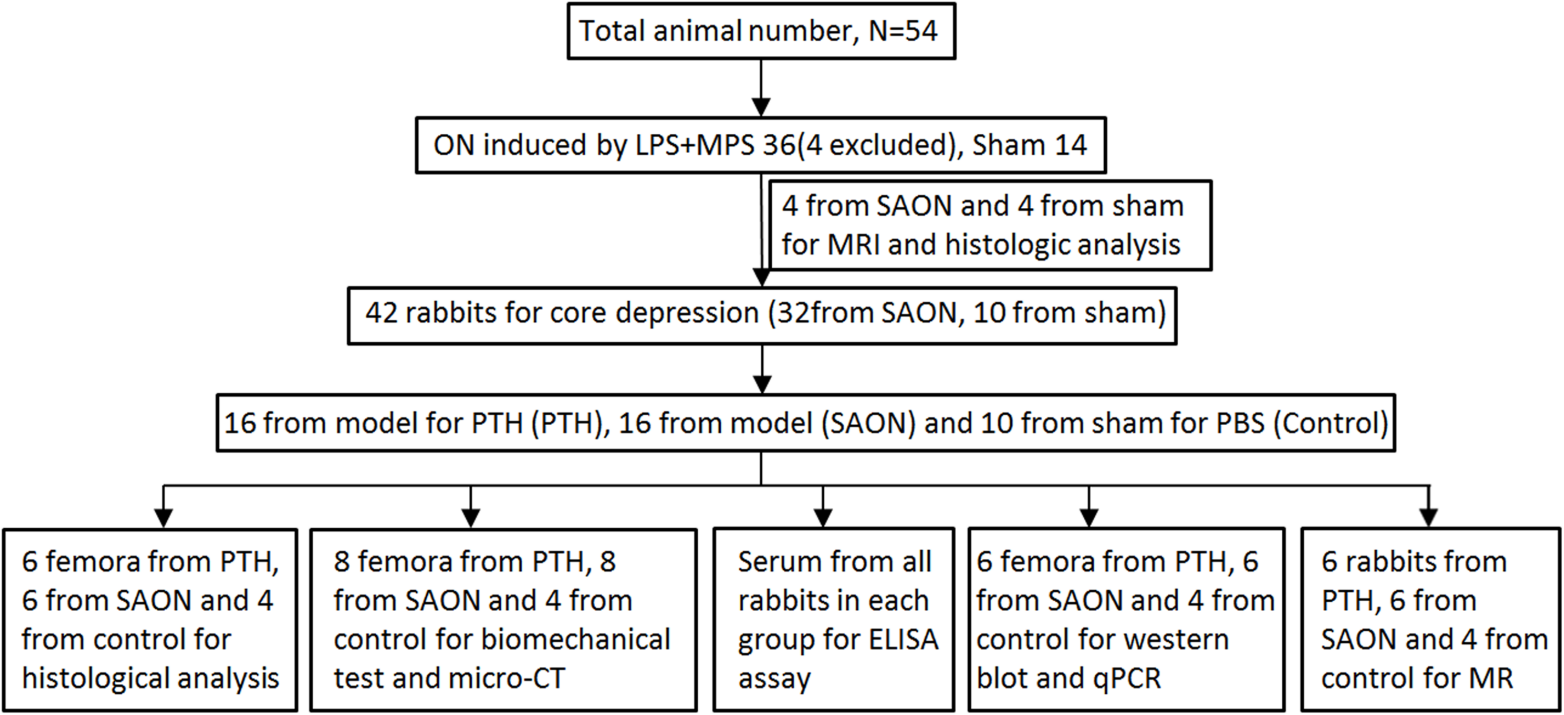
Study design and number of animals utilized at different stages. ON = osteonecrosis; LPS = lipopolysaccharide; MPS = methylprednisolone; SAON = steroid-associated osteonecrosis.

Fifty-four adult male New Zealand White Rabbits (2.82±0.16 kg) were used, including 40 for the osteonecrosis group (SAON) and 14 for the sham group (sham) (Fig 1). Necrosis was induced by administration of steroids according to our previously reported protocol [19]. Briefly, one pinna marginal intravenous injection of 10 μg/kg lipopolysaccharide (LPS, Escherichia coli 0111:B4; Sigma, USA) was administered to the rabbits in the SAON group, followed by three continuous intramuscular injections of methylprednisolone (20 mg/kg, MPS, Pharmacia and Upjohn, Puurs, Belgium) at 24-hour intervals. The sham group received NS as control. Four rabbits were humanely euthanized under the guidance of vet, due to reduced food intake and more than 10% acute weight loss after LPS and MPS injection. As reported previously, osteonecrosis lesions formation occurs 2 weeks after methylprednisolone injection [20, 21]. To confirm the occurrence of osteonecrosis, four rabbits each in the SAON and the sham groups were randomly selected for MRI on day 0 and day 14, and were sacrificed for histological analysis after MRI scanning.

### Core decompression and PTH treatment

**Fig 2:**
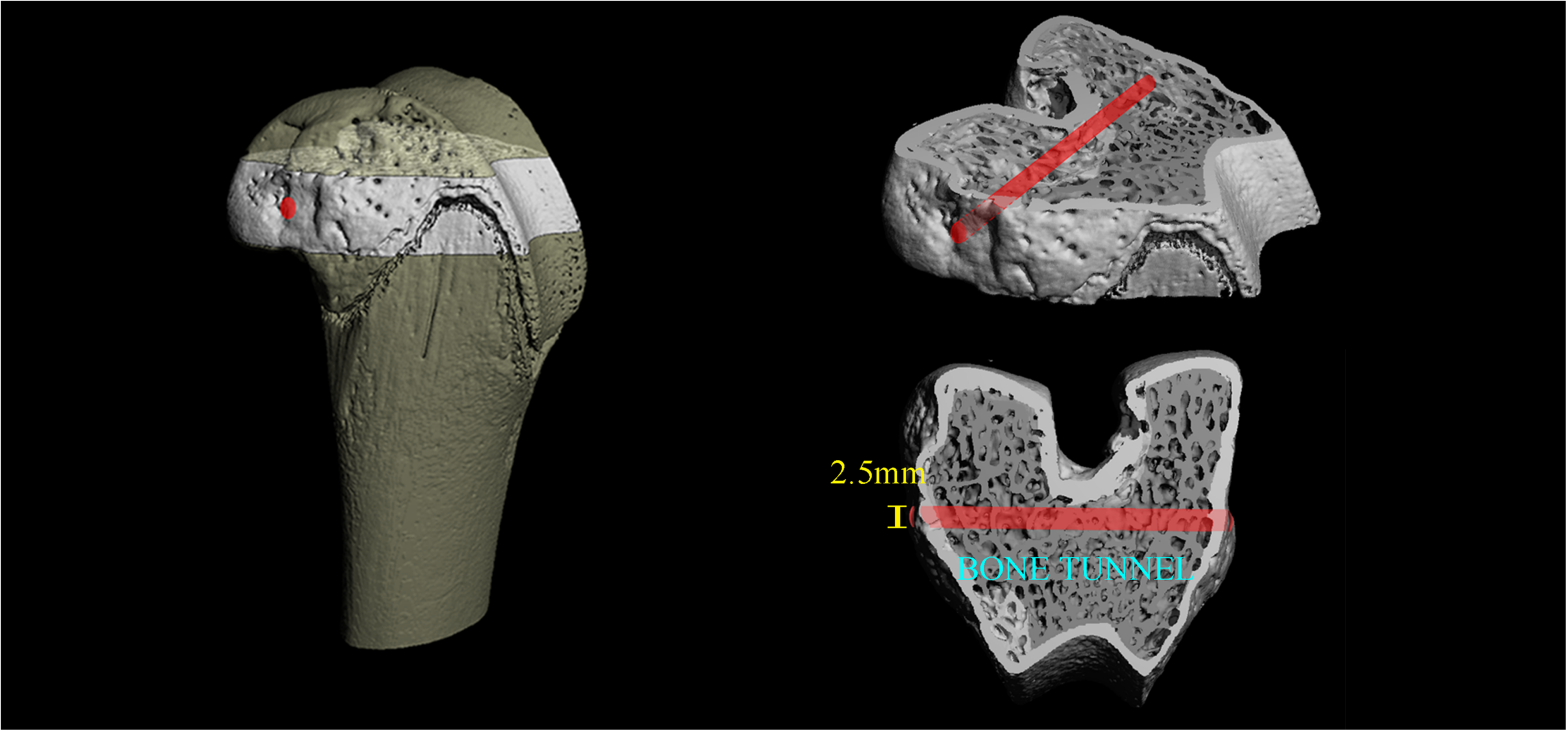
Schematic diagram shows the creation of bone tunnel by CD in the distal femur of rabbits. CD was performed bilaterally from the attachment of the medial collateral ligament to the contralateral cortex at the medial aspect of the distal femur by drilling a 2.5-mm diameter tunnel.

All surviving rabbits were anaesthetized with intramuscular xylazine (2 mg/kg) and ketamine (50 mg/kg). CD was performed bilaterally from the attachment of the medial collateral ligament to the contralateral cortex at the medial aspect of the distal femur by drilling a 2.5-mm diameter hole as described previously (Fig 2) [10, 22]. Rabbits in the SAON group were equally divided into the SAON-CD and SAON-CD-PTH groups randomly (n = 16 each) while the sham group served as the Sham-CD group (n = 10). Animals in the SAON-CD-PTH group were administered recombinant PTH [1–34] (30 μg/kg/day, Bachem, Bubendorf, Switzerland) from day 1 to week 6 postoperatively while the other two groups received NS. Body weights were measured to adjust the drug dosages. Buprenorphine (0.3mg/kg/SC) was used pre-operation while Carprofen (4mg/kg/PO) was used post-operation for analgesia and Penicillin G Procaine (30000U/lb./IM) was used to prevent infection in the first three days after operation. No animal was found ill or dead post-operatively. Calcein green (5 mg/kg; sigma) and alizarin red (30 mg/kg) injections were performed 14 and 4 days before sacrifice, respectively. All animals were euthanized after six-weeks of PTH therapy for further analyses (Fig 1).

### MR imaging and analysis

Rabbits were anesthetized with intramuscular xylazine (2 mg/kg) and ketamine (50 mg/kg) and MRI was performed (T1-weighted image, TR 540ms, TE 10 ms; T2-weighted image, TR 2500ms, TE 120ms) with a 3.0-T superconductive unit (General Electric Medical Systems, Milwaukee, WI) on Day 0 (baseline measure), Day 14 and Day 56 after LPS and MPS injection [19]. Four rabbits were randomly selected in each group for MRI on Day 0, 14, and 56.

To assess MR changes in femoral bone marrow, the region of interest (ROI) was set between the distal metaphysis and distal diaphysis. The average signal intensity (SI) of ROI on T1- and T2-weight images and the area ratio of abnormal SI in the femoral marrow were quantified using Image-Pro Plus 6.0 software (Media Cybernetics Inc, Maryland, USA). The percentage of enhancement ratios (ER) was calculated according to the following formula: ER = SI/ SI_Day0_ *100%. Area of abnormal SI (AASI) was calculated according the following equation: AASI/Area of bone marrow [23, 24].

### Micro-CT scan and quantitative analysis

Distal femurs (n = 4 in the Sham-CD group, n = 8 in the SAON-CD group, and n = 8 in the SAON-CD-PTH group) were subjected to micro-CT scanning with an isometric resolution of 34.4 μm, using a Scanco μCT100 instrument (Scanco Medical, Bassersdorf, Switzerland). One-hundred and sixty slices of the bone tunnel were obtained by three-dimensional (3D) image reconstructions. To characterize the newly formed bone, the mean volumetric bone mineral density (vBMD, mg/cm3), bone tissue volume density (BV/TV, %), connectivity density (Conn.D, 1 mm3), trabecular number (Tb.N, 1 mm), trabecular separation (Tb.Sp, mm), and structure model index (SMI) were measured within the bone tunnel.

### Biomechanical evaluation

To evaluate the healing quality of new bone, the specimens were subjected to a compression test with a small-diameter (2.5 mm) indenter after positioning the tunnel.[10] The distal femurs were prepared to an identical thickness of 8.5 mm with the bone tunnel in the middle. Specimens were tested with a Zwick/Roell 2.5 material testing system (Zwick, Ulm, Germany). A load was applied to compress the bone precisely perpendicular to the middle of the bone tunnel at a rate of 10mm/min (S1 Fig). The TestXpertⅡsoftware (Zwick/Roell) was used to record stiffness (N/mm) and maximum load (N).

### Histological and immunohistochemical analyses

Specimens were bisected along the sagittal plane into two parts for decalcified and undecalcified histological analysis, respectively. After decalcification in 10% (w/v) ethylenediaminetetraacetic acid (EDTA), the lateral halves (n = 4 in the Sham-CD group, n = 6 in the SAON-CD group, n = 6 in the SAON-CD-PTH group) were dehydrated and cut into five-micron thick sections perpendicular to the longitudinal axis of the bone tunnel. Subsequently, the sections were stained with hematoxylin and eosin (H&E staining) for evaluating necrosis, Toluidine Blue staining to count the osteoblasts and osteoclasts, and α-SMA (Abcam, Hong Kong) to detect blood vessels. The medial halves were embedded in resin without decalcification and cut into 10-μm sections.

The decalcified sections were analyzed under light microscopy (Olympus BX51, Tokyo, Japan) and undecalcified ones were detected using fluorescence microscopy (Leica DM5 500B, Leica Microsystems, Bensheim, Germany). Bone marrow cell necrosis, empty osteocyte lacunae and any evidence of bone repair are the histological characteristics of osteonecrosis [18, 25]. To evaluate the efficacy of treatment, the parameters of blood vessels, empty osteocyte lacunae, necrosis foci, MS/BS and MAR were analyzed as described previously [19, 26]. Five random fields within each ROI were selected and analyzed with the Image-Pro Plus 6.0 software.

### RNA isolation and quantitative PCR

Distal femurs (n = 4 in the Sham-CD group, n = 6 in the SAON-CD group, n = 6 in the SAON-CD-PTH group) were cut into half and crushed into powder with liquid nitrogen. Total RNA was extracted using the Qiagen RNeasy Mini kit (Qiagen, Valencia, CA, USA), and reverse-transcribed into complementary DNA (cDNA) using a Double-Strand cDNA Synthesis Kit (Takara, Dalian, China) according to the manufacturers’ instructions. The PCR reaction was performed using the Power SYBR® Green PCR Master Mix (Takara) with 1μL of cDNA as template on the ABI StepOnePlus System (Applied Biosystems, Warrington, UK). The cycling conditions were as follows: 95°C for 30 s and then 40 cycles of 95°C for 5 s and 60°C for 30 s. *18s* was utilized as the housekeeping gene and the relative quantity of mRNA was calculated (2^−ΔΔCt^ analysis). The primer sequences were as follows: *Runx2*: forward, 5’-CAGTCTTACCCCTCTTACC-3’ and reverse, 5’-CATCTTTACCTGAAATGCG-3’; *BMP2*: forward, 5’-GGACGACATCCTGAGCGAGT-3’ and reverse, 5’-CGGCGGTACAAGTCCAGCAT-3’; *VEGF*: forward, 5’-GGGGGCTGCTGCAATGATGAAA-3’ and reverse, 5’-GCTGGCCCTGGTGAGGTTTGAT-3’; *bFGF* (basic fibroblast growth factor): forward, 5’-GTGCAAACCGTTACCTTGCT-3’ and reverse, 5’-ACTGCCAGTTCGTTTCAGT-3’; *IGF-1*(insulin-like growth factor-I): forward, 5’- TGGTGGATGCTCTCAGTTCGTGT-3’ and reverse, 5’- GCTGATACTTCTGAGTCTTGGGCA-3’; *18s*: forward, 5’-GACGGACCAGAGCGAAAGC-3’ and reverse, 5’-CGCCAGTCGGCATCGTTTATG-3’.

### Western blot

Distal femurs (n = 4 in the Sham-CD group, n = 6 in the SAON-CD group, n = 6 in the SAON-CD-PTH group) were lysed in radioimmunoprecipitation assay (RIPA) buffer. Total protein were separated on SDS polyacrylamide gels and transferred to membranes (Bio-Rad, Hercules, USA). After non-specific blocking, membranes were incubated with primary antibodies, followed with appropriate secondary antibodies. After rinsing, the blots were visualized with SuperSignal Chemiluminescent substrate (Thermo Scientific, Rockford, IL, USA). The following primary antibodies were utilized: anti-BMP2, anti-VEGF (1:200; Santa Cruz Biltech, Inc, Santa Cruz, CA, USA) and anti-tubulin (1:8000, Sigma-Aldrich, USA).

### Serum biomarkers

Prior to storage at -80°C, blood was collected during euthanasia through the common iliac artery and centrifuged to separate serum at 2,000 rpm (425xg) for 5 min at 4°C as described previously [27, 28]. Serum OCN and VEGF levels were analyzed by using rabbit-specific enzyme-linked immunosorbent assay kits (Bioleaf Biotech, Shanghai, China) according to the manufacturer's protocol. The serum calcium level was measured by the automatic biochemical analyzer (Beckman Coulter Au5400, Beckman Coulter Inc., Brea, CA, USA). All samples, diluted appropriately, were assayed three times.

### Statistical analysis

All data were expressed as mean ± standard deviation (SD). Statistical analysis was performed with SPSS 16.0 software (SPSS, Chicago, IL, USA). Differences in unpaired data between groups were analyzed by the Student’s two-tail t-test after confirming normality by the Kolmogorov-Smirnov test. The nonparametric Mann-Whitney test was conducted if the data did not follow the normal distribution. One-way ANOVA and Kruskal–Wallis test were used to analyze the differences between multiple data sets. Statistically significant differences with repeated variables among different positions and treatment were evaluated by two-way ANOVA. The threshold of statistical significance was set at p < 0.05.

## Results

### Validation of the SAON model

**Fig 3:**
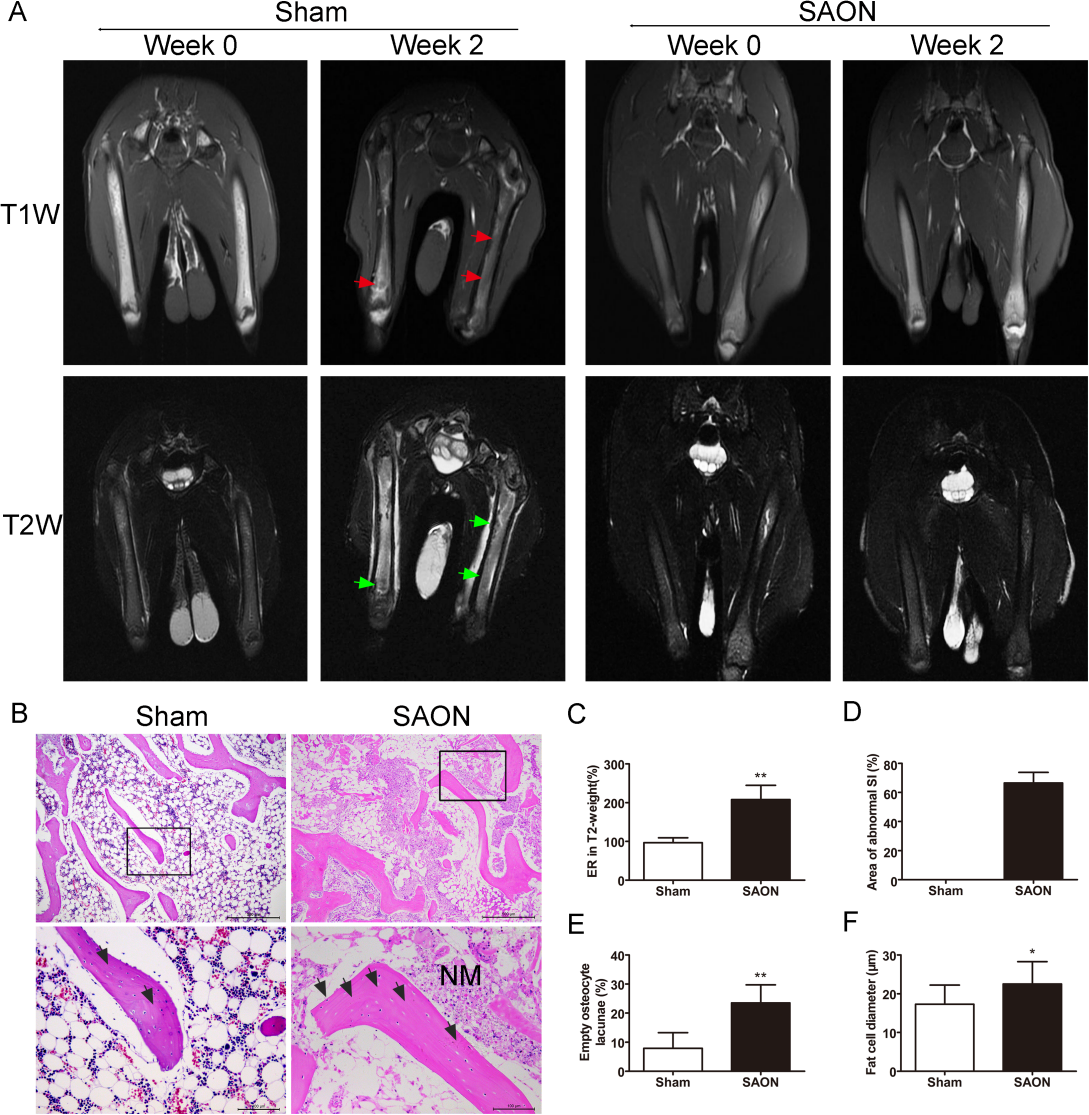
MRI images and histological analysis confirmed successful establishment of the SAON model. (A) MRI images of femurs on T1 and T2 weight from the SAON group (n = 4) and the Sham group before and 2 weeks after steroid administration, exhibited diffused and decreased SI (red arrows) in T1W. Focal hypointense lesion (green arrows) was surrounded by diffuse hyperintense area in T2W at week 2, but no abnormal signal was found at week 0. MRI images of femurs in T1 and T2 weight from the sham group (n = 4) were obtained at week 0 and week 2 after vehicle injection, and displayed no abnormal SI. (B) Histological analyses of distal femurs from each group (n = 4). H&E staining after steroid-induced osteonecrosis showed necrotic mass, enlarged fat cells and massive empty osteocyte lacunae (black arrows) in the SAON group. The ER on T2 weight MR images (C) and the area of abnormal SI (D) were measured for the quantification of MR images. The percentage of empty osteocyte lacunae (E) and the mean fat cell diameter (F) were measured. Data are presented as mean ± SD, and error bars in the figure denote SD, **p<0.01,*p<0.05.

Four rabbits were excluded because of anesthetic accident and infection. Four animals each in the SAON and the sham groups were randomly selected for MRI and histological analysis. Fig 3 showed that the appearance of abnormal SI, increased ER on T2-weight and decreased ER on T1-weight. This confirmed that osteonecrosis in the distal femurs was established successfully, while no changes were observed in the sham group. The remaining animals were euthanized after six-weeks of PTH/normal saline (NS) therapy.

### PTH improves the efficacy of CD in the treatment of SAON

**Fig 4:**
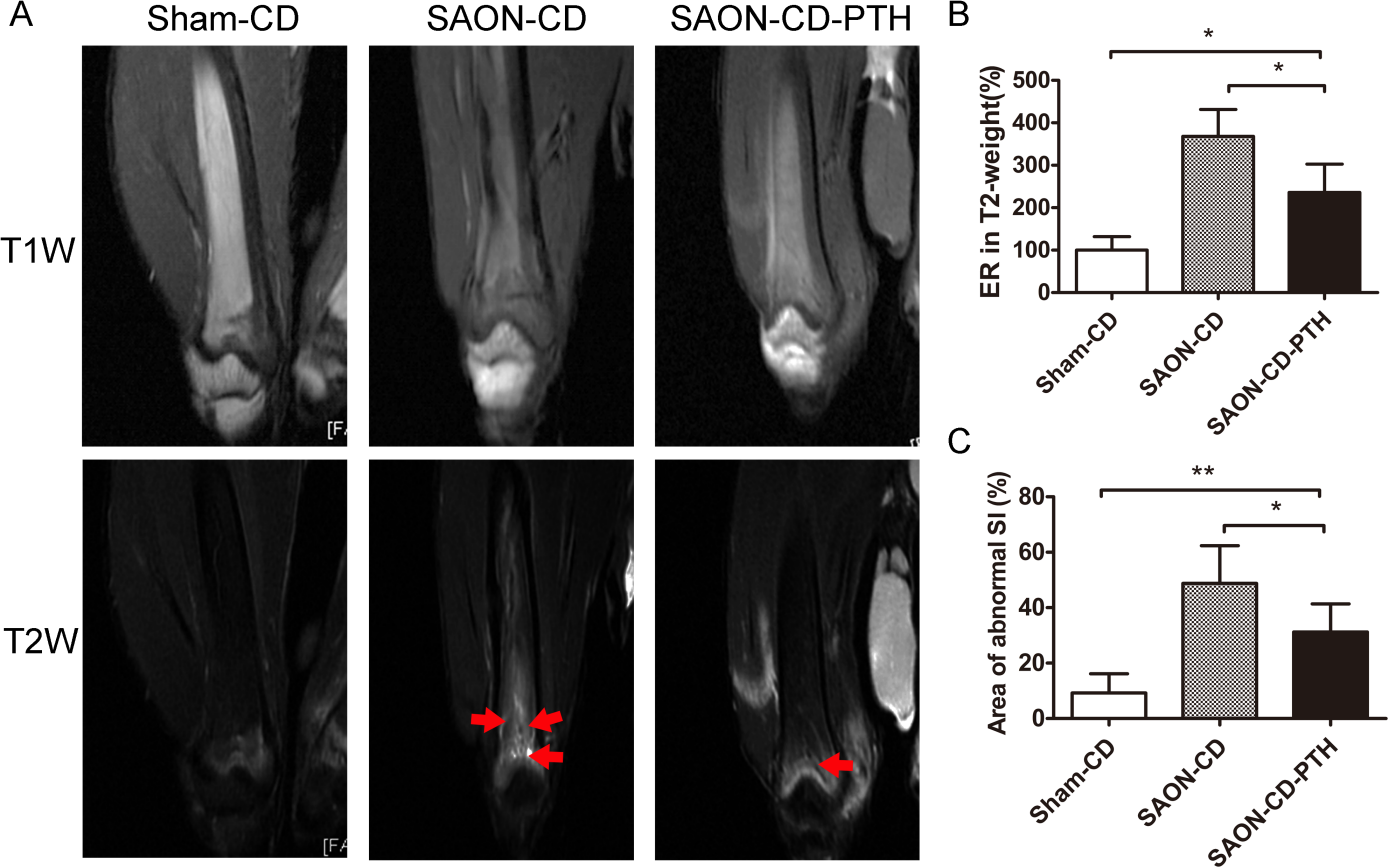
PTH improves the efficacy of CD in the treatment of SAON. (A) MR images of the distal femurs from each group (n = 4 in the Sham-CD group, n = 6 in the SAON-CD group and n = 6 in the SAON-CD-PTH group) showed normal signal intensity in the Sham-CD group. Focal inhomogeneous high SI in the T2-weight images (red arrows) and focal inhomogeneous low SI in the T1-weight in the SAON group. However, in the SAON-CD-PTH group, less high SI in the T2 weight image and less abnormal SI in the T1 weight image were revealed the ER in the T2 weight MR images (B) and the area of abnormal SI (C) were measured for the quantification of MR images.

As shown in Fig 4A, a normal signal was demonstrated in distal femurs of the Sham-CD group, with homogeneous high SI on the T1-weight image and low SI on the T2-weight image. However, in the SAON-CD group, there was focal inhomogeneous high SI on the T2-weight images and focal inhomogeneous low SI in the T1-weight image. Less abnormal SI on both T1-weight and T2-weight was observed in the SAON-CD-PTH group. The quantification of the area of abnormal SI and ER in the T2-weight image (Fig 4B and 4C) further confirmed the results. Collectively, the MR results demonstrated that PTH administration improved the repair of CD in rabbits with SAON.

### PTH enhances osteogenesis in/out of the tunnel in the distal femur

**Fig 5:**
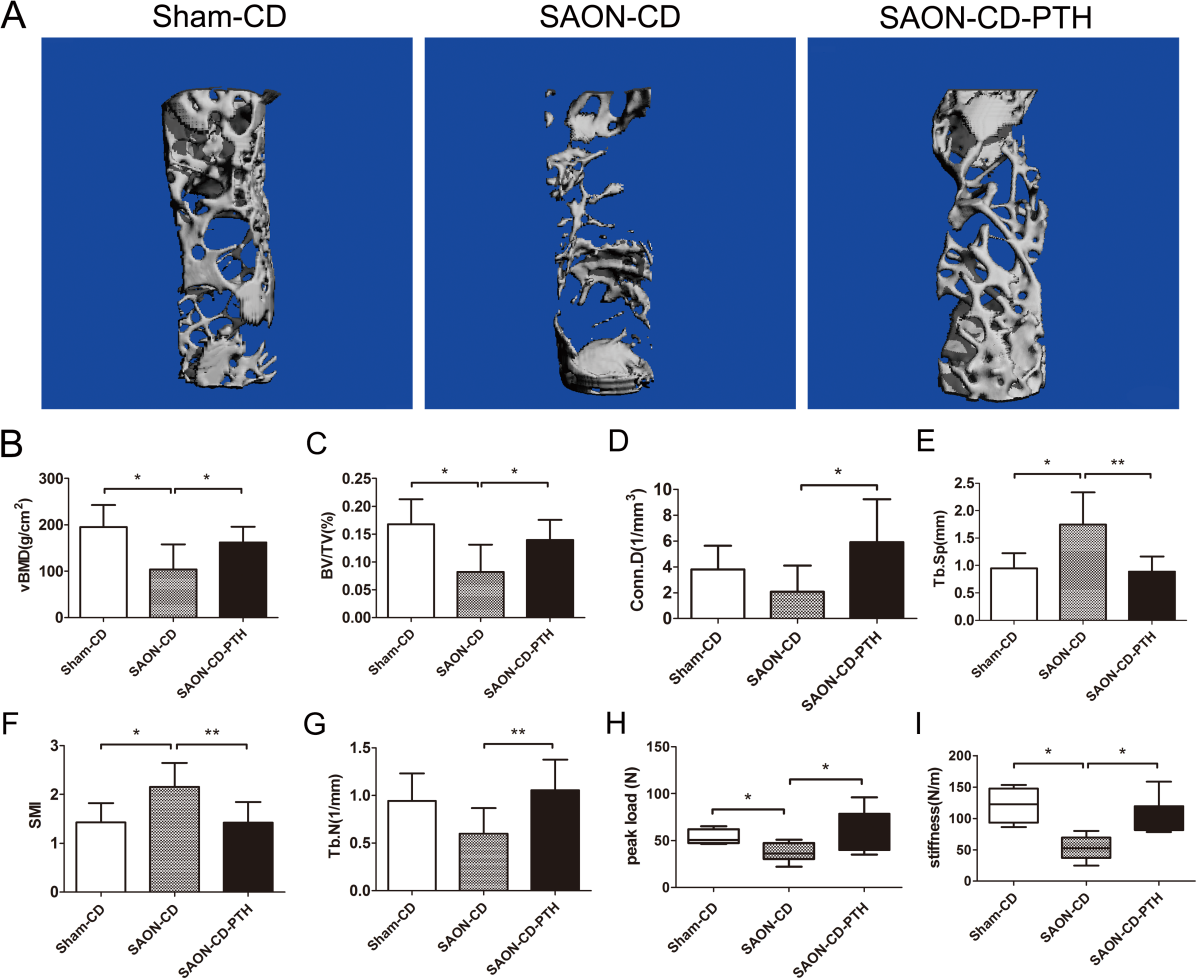
Representative 3D micro-CT images and biomechanical compression test showed that PTH enhanced osteogenesis in the tunnel of CD. (A) Representative 3D micro-CT images reveal new bone formation within the tunnel of each group at 6 weeks after CD. (B-G) Quantitative micro-CT show the mean volumetric bone mineral density (vBMD), bone tissue volume density, connective density, trabecular number, trabecular separation, and structure model index. (H-I) Peak load and stiffness of the bone tunnels from each group are presented. n = 8 in the SAON-CD-PTH group, n = 8 in the SAON-CD group, n = 4 in the sham-CD group. Data are presented as mean ± SD, and error bars in the figure denote SD, *p<0.05, **p<0.01.

Three-dimensional micro-CT images (Fig 5A) revealed less bone formation within the bone tunnel in the SAON-CD group, as evidenced by decreased values of BMD and BV/TV, together with increased values of SMI and Tb.Sp. In the PTH-treated group, bone formation was significantly enhanced and the microstructural indices are shown in Fig 5A. Compared with the SAON-CD group, the vBMD (162.46±31.27 vs. 103.98±50.57mg/cc, P = 0.021), the BV/TV (13.97±3.37 vs. 8.22±4.56%, P = 0.018), the Conn.D (5.91±3.09 vs. 2.09±1.88/mm3, P = 0.014) and Tb.N (1.05±0.30 vs 0.60±0.25, P = 0.08) were increased, but the SMI (1.42±0.4 vs 2.05±0.46, P = 0.006) and Tb.Sp (0.89±0.26 vs. 1.75±0.55 mm, P = 0.002) were markedly decreased in the SAON-CD-PTH group.

After micro-CT scanning, the specimens were subjected to compression tests. Decreased maximal load (53.18±7.21 vs.37.37±9.27 N, P = 0.033) and stiffness (121.21±24.52 VS.52.65±18.07 N/m, P = 0.002) were detected in the SAON-CD group, as compared to the Sham-CD group (Fig 5H and 5I). In contrast, both the loads to failure (60.40±20.45 vs.37.37±9.27 N, P = 0.045) and stiffness (100.40±27.56 VS. 52.65±18.07 N/m, P = 0.009) were enhanced upon treatment with PTH (Fig 5H and 5I).

**Fig 6:**
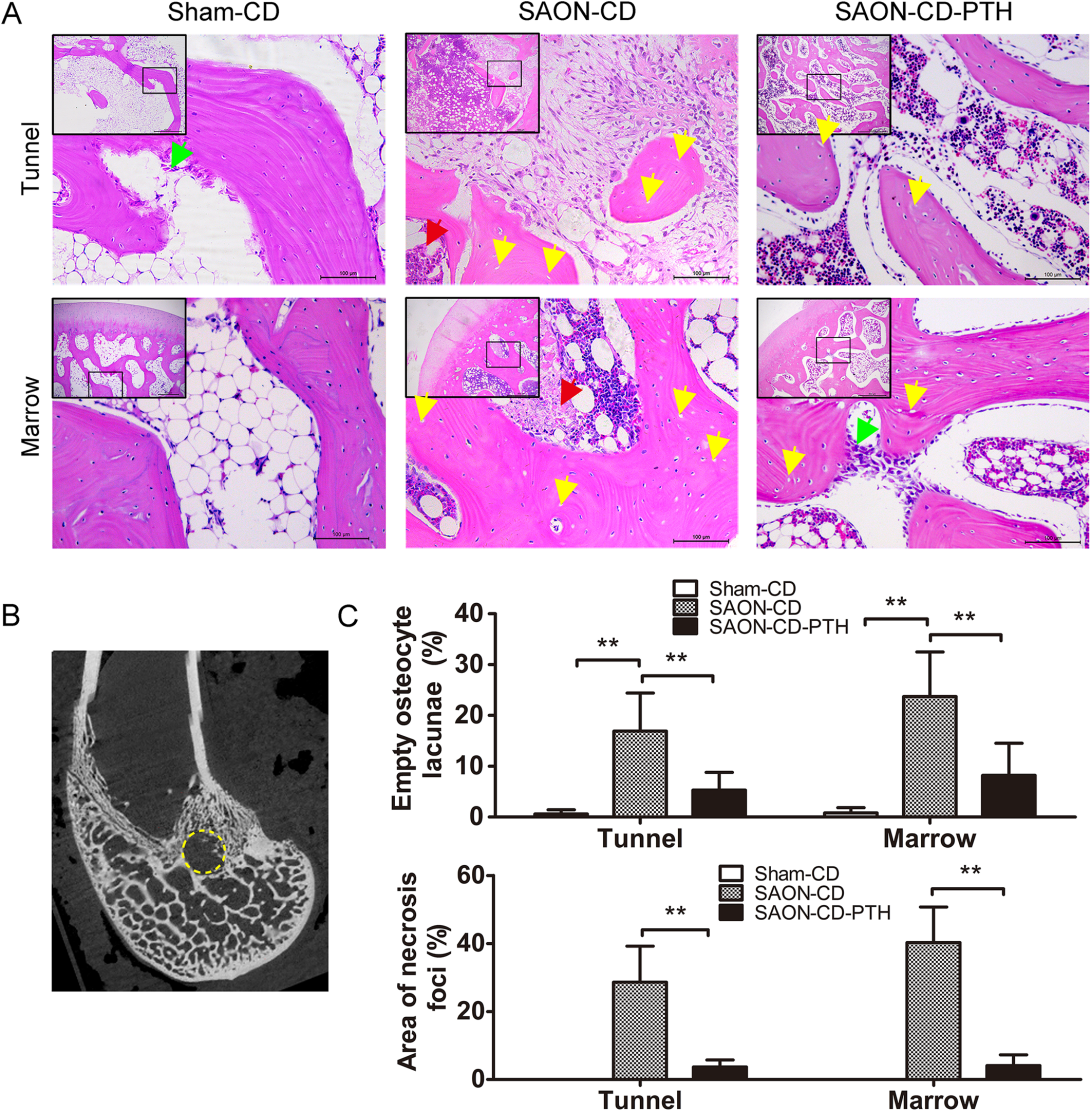
PTH promotes the repair of SAON after CD surgery in the bone tunnel and bone marrow of each group. (A) PTH enhanced bone formation, increased the number of osteoblasts (green arrows), reduced necrosis foci (red arrows), and decreased the number of mononuclear cells and empty lacunas (yellow arrows) in the distal femurs with steroid-induced osteonecrosis after CD. (B) in the coronal plane of distal femur with a bone tunnel (yellow dotted circles). (C) The percentage of the area of necrosis foci and the percentage of empty osteocyte lacuna were measured, n = 6 in the SAON-CD-PTH group, n = 6 in the SAON-CD group, and n = 4 in the sham-CD group, Data are presented as mean ± SD, and error bars in the figure denote SD, *p<0.05, **p<0.01 (H&E, 50x and 200x).

Histological analysis (Fig 6A) revealed normal bone marrow and newly formed bone in the tunnels of the Sham-CD group while those in the SAON-CD group were filled with abundant fibrous tissue and massive empty lacunas, as well as some osteoclasts, and fewer osteocytes and osteoblasts. However, enhanced bone formation was observed in the SAON-CD-PTH group, as compared to the SAON-CD group, with a marked reduction of empty lacunas and fibrous tissue and an increase in the number of osteocytes and osteoblasts (Fig 6C and S2A Fig). The quantification of area of necrosis foci and empty osteocyte lacunae, showed that SAON could increase the area of necrosis foci and the percentage of empty osteocytes lacunae while PTH treatment could obviously reduce these parameters (P<0.01, respectively).

**Fig 7:**
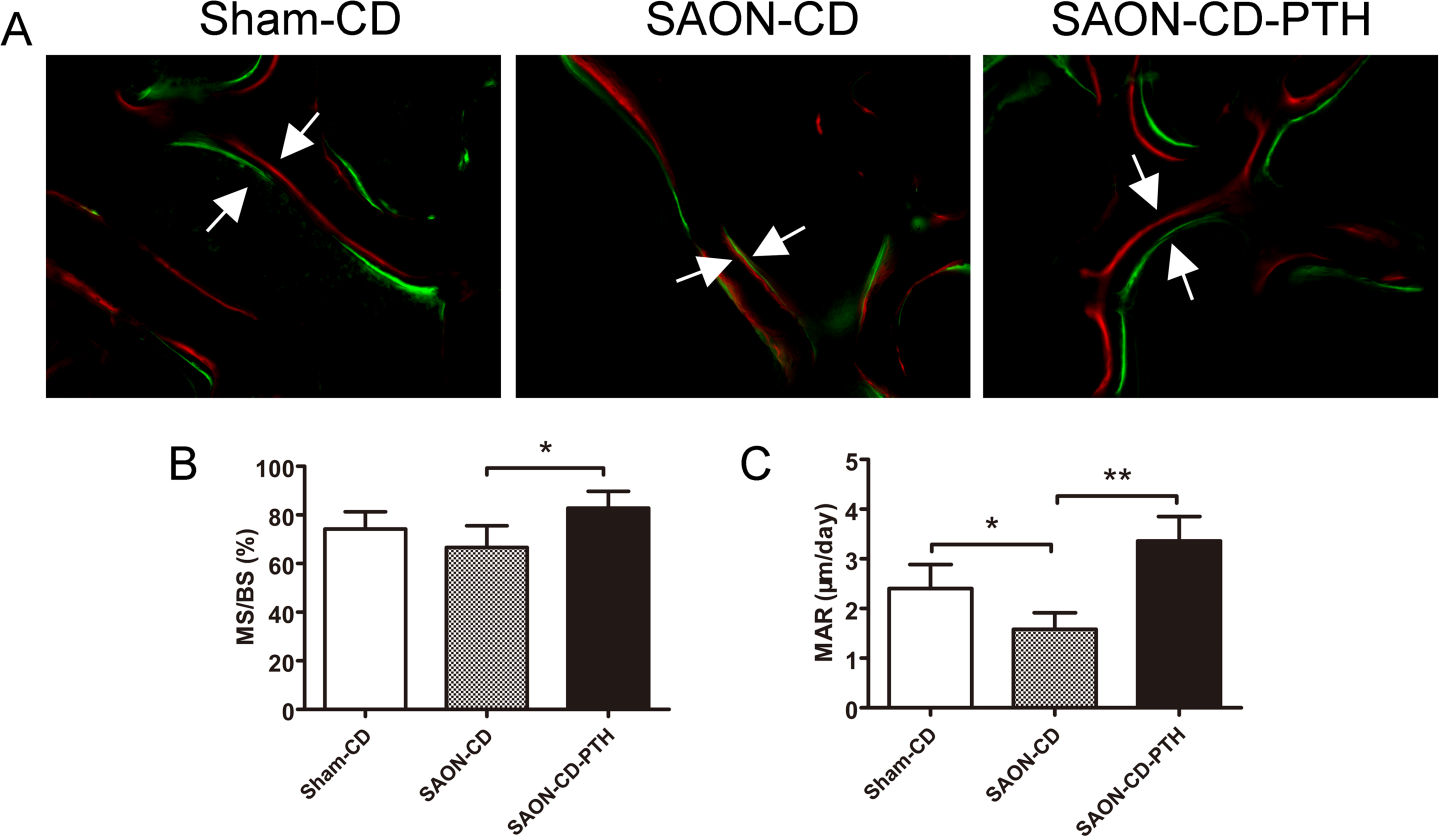
PTH upregulates bone formation in SAON after CD surgery. Bone was labeled during regeneration and remodeling by calcein (green) and andalizarin red (red) (A). (B-C) The MS/BS and the MAR were measured and presented. Data are presented as mean ± SD, and error bars in the figure denote SD, *p<0.05, **p<0.01.

The bone formation out of the tunnels in the marrow is shown in Fig 6A as well. No abnormal changes were observed in the Sham-CD group. However, in the SAON-CD group, a disordered architecture of marrow tissue was observed with osteonecrosis foci, massive empty lacunae and mononuclear cells being present in the bone, with few osteoblasts lining the trabecular bone. Compared with the SAON-CD group, PTH could markedly enhance bone formation with more osteoblasts. No osteonecrosis foci, and fewer empty lacunae were found in the bone tissue. The quantitative data are shown including decreasing necrosis foci and empty osteocyte lacunae (P<0.01, respectively). Moreover, in the fluorescent analysis (Fig 7A–7C), specimens from the SAON-CD group exhibited a lower mineral apposition rate (MAR) and mineralizing surface/bone surface (MS/BS), compared with the sham-CD group, even though no statistically significant differences were found in MS/BS. Administration of PTH exerted a strong effect on bone mineralization, as evidenced by improved MAR and MS/BS.

### PTH enhances neovascularization in the CD tunnel and bone marrow

**Fig 8:**
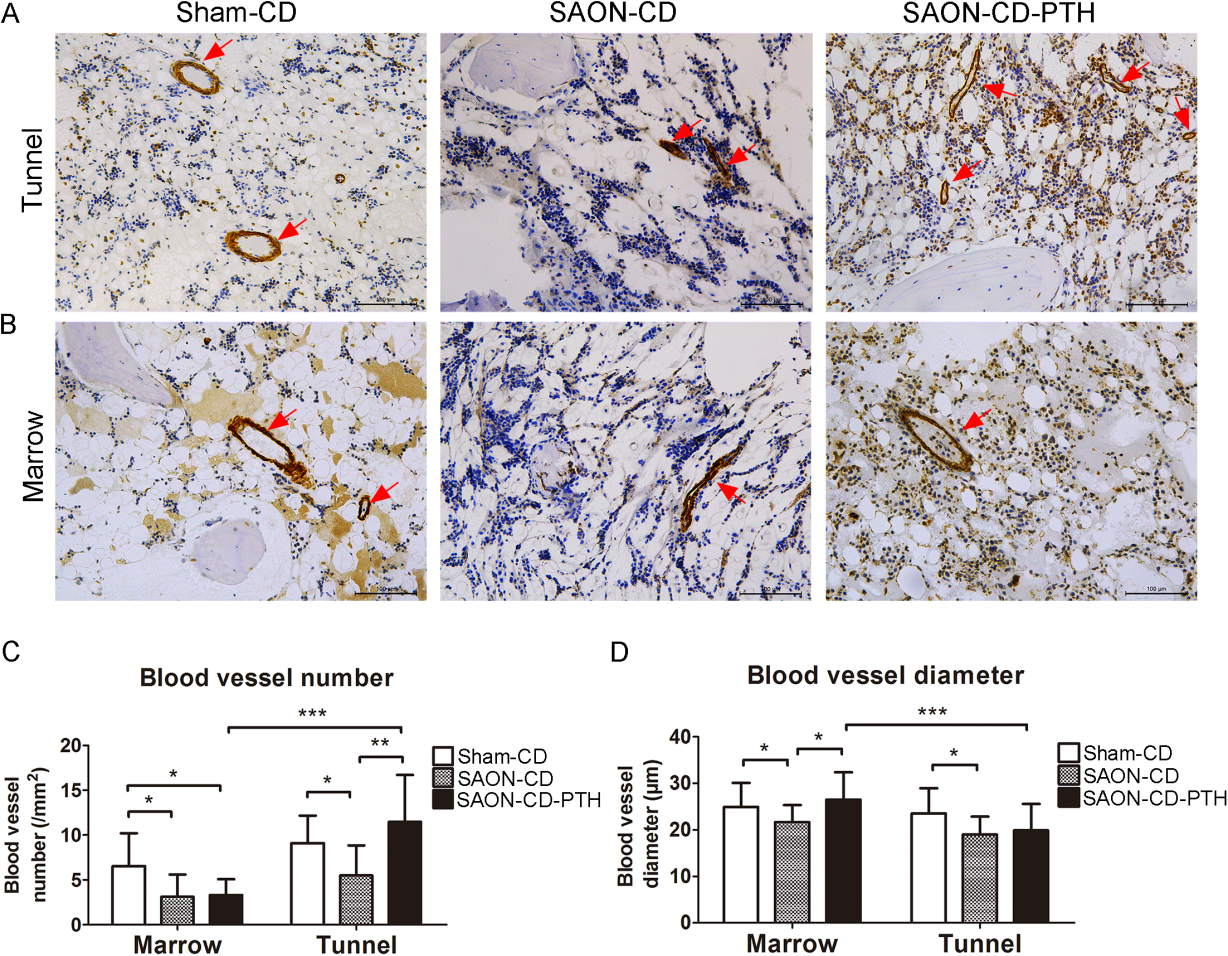
PTH improves revascularization both in the tunnel and in the bone marrow. Vessels (red arrows) from each group (n = 6 in the SAON-CD-PTH group, n = 6 in the SAON-CD group, and n = 4 in the sham-CD group) were visualized with α-SMA staining (200x) within the tunnel (A) and in the marrow (B). (C) The number per area of tissue and (D) the diameter of blood vessels were measured. Data are presented as mean ± SD, and error bars in the figure denote SD, *p<0.05, **p<0.01.

The α-smooth muscle actin (α-SMA) staining was used to evaluate the effects of PTH on neovascularization within the CD tunnel. The vessels in the SAON-CD group displayed flattened shape (Fig 8A) with smaller diameter (decreased by 39.6%) and number (decreased by 19.0%) compared with the Sham-CD group (P<0.05, respectively) (Fig 8C). More vessels were found (P<0.01) within the CD tunnel in the PTH treatment group (11.46±4.98/mm^2^) compared with the SAON-CD group (5.50±3.19/mm^2^), while there was no significant difference in the mean diameters of vessels (3.12±2.36 vs.3.30±1.69/mm^2^, P>0.05) between the two groups (Fig 8D).

As CD could reduce bone marrow pressure and improve capillary blood flow [8], we also evaluated neovascularization in the bone marrow. Fig 8B–8D showed that the number and the mean diameter of vessels in the bone marrow were decreased in the SAON-CD group when compared to the Sham-CD group (P<0.05). Interestingly, the mean diameter of blood vessels in the bone marrow increased in the SAON-CD-PTH group (21.67±3.57 vs.26.51±5.70μm, P<0.05) while there was no significant difference in vessel numbers compared to the SAON-CD group (19.06±3.67 vs.19.89±5.52μm, P>0.05).

### PTH enhances local and systemic expression of osteogenesis and neovascularization markers

**Fig 9:**
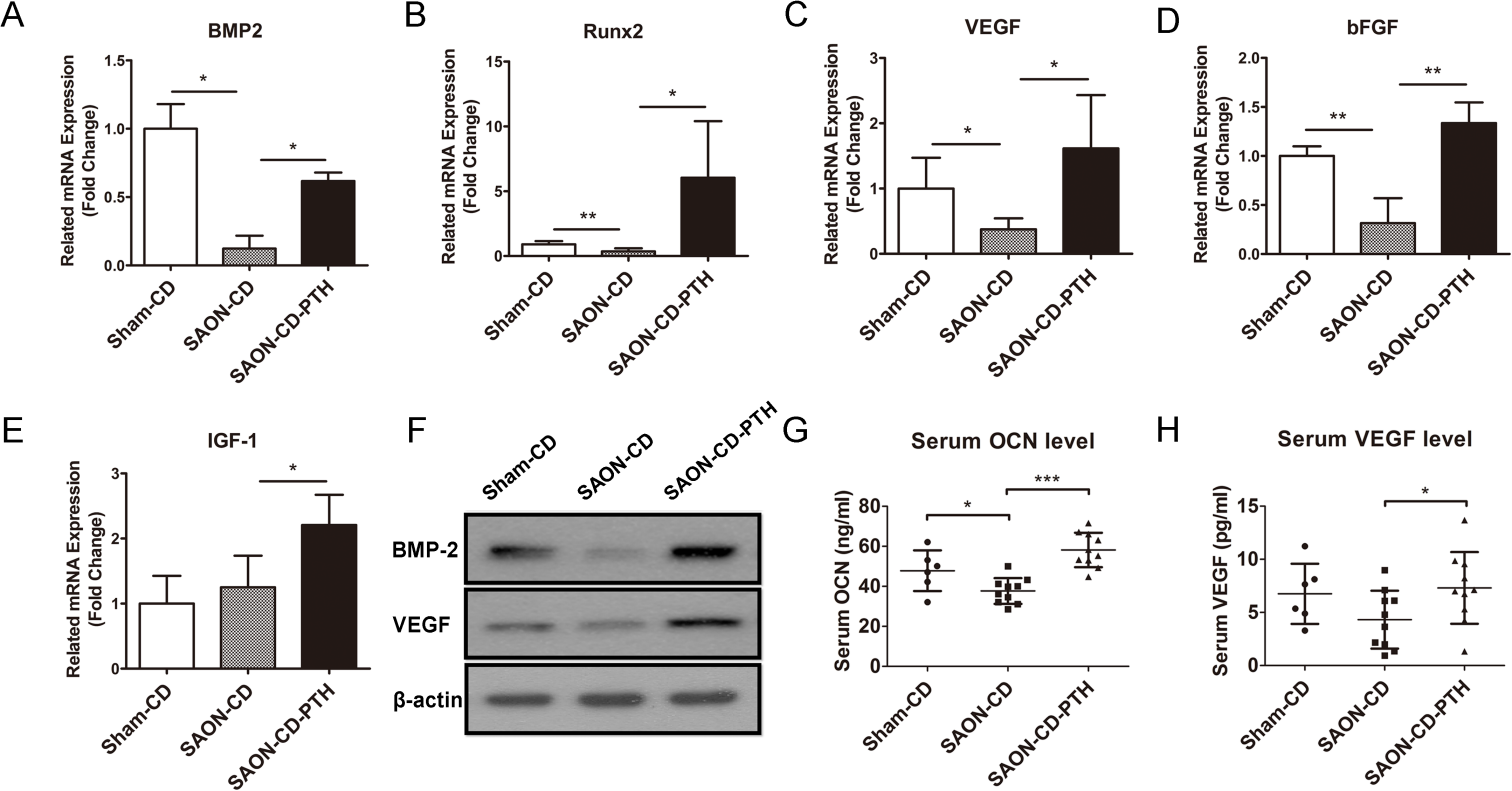
PTH enhances local and systemic expression of osteogenesis and neovascularization markers. (A-E) RT-PCR detected the gene expression levels of BMP2, Runx2, IGF-1. bFGF and VEGF in the distal femurs. (n = 6 in the SAON-CD-PTH group, n = 6 in the SAON-CD group, and n = 4 in the sham-CD group). (F) Western blot detected BMP-2 and VEGF expression in the distal femurs from each group (n = 6 in the SAON-CD-PTH group, n = 6 in the SAON-CD group, and n = 4 in the sham-CD group). ELISA of serum concentration of (G) osteocalcin (OCN) and (H) VEGF from each group (n = 10 in the SAON-CD-PTH group, n = 10 in the SAON-CD group, and n = 6 in the sham-CD group) were measured. Data are presented as mean ± SD, and error bars in the figure denote SD, *p<0.05, **p<0.01.

To explore the mechanisms of osteogenesis and neovascularization during SAON repair, we analyzed the expression of genes and proteins that PTH[1–34] can potentially affect in the downstream signaling pathways. *IGF-1*, *bFGF*, *BMP2* and *RUNX2* are the downstream osteogenic genes modulated by PTH[1–34] in promoting bone formation, osteogenic differentiation and increased osteoblast activity [29–31], while VEGF is regarded as a marker of neovascularization [32]. As shown in Fig 9A–9F, the mRNA and protein expression levels of *BMP-2*, *bFGF*, *RUNX2* and *VEGF* were decreased while the mRNA expression of IGF-1 was elevated. There were no significant differences between the SAON-CD group versus Sham-CD group. However, a significant increase of mRNA and/or protein expression levels of *BMP-2*, *bFGF*, *RUNX2*, *IGF* and *VEGF* were observed in the SAON-CD-PTH group versus the SAON-CD group.

Serum osteocalcin (OCN), a marker of bone formation, was reduced by 17.32% in the SAON-CD group compared to the Sham-CD group. Nevertheless, PTH treatment increased serum OCN levels which were reduced in the SAON-CD group, to a higher degree (Fig 9E). A higher serum calcium level was observed in the SAON-CD-PTH group versus the Sham-CD and SAON-CD-PTH group (S3 Fig). A significant increase (P<0.05) in serum VEGF level, a marker of vascularization reported previously [33, 34], was detected with PTH treatment, as compared to the SAON-CD group (Fig 9F).

## Discussion

SAON often leads to collapse, which require joint arthroplasty in the final stages [35]. CD is considered an effective therapy for early-stage osteonecrosis, but the prognosis is rather poor. Various adjunctive therapies to improve the therapeutic effects of CD have been attempted, but none of these are fully effective. Therefore, an alternative therapeutic strategy is required. This study demonstrated that: (1) PTH[1–34] improved the efficacy of CD in the treatment of SAON; (2) PTH[1–34] enhanced the effects of CD on osteogenesis under steroid administration, by increasing the number of osteoblasts, decreasing the number of necrosis foci and empty lacunae, thereby resulting in better biomechanical properties; (3) Neovascularization was improved by PTH[1–34] administration after CD by increasing blood vessel number in the bone tunnel and enlarging the diameter of vessels in the bone marrow; (4) PTH[1–34] upregulated the expression of BMP-2 and VEGF protein in distal femurs, as well as increased serum OCN and VEGF levels.

Nevertheless, there are some limitations to our study. Although the rabbit model of SAON has been widely utilized with a high incidence of necrosis [10, 19, 22], no joint collapse caused by ON lesions occurs. This is attributed to differences in weightbearing, particularly at the hip, between quadrupedal animals and bipedal humans. Moreover, the exact mechanism by which the combination of PTH and CD improves treatment efficacy is still unclear, thus warranting further studies.

MRI is commonly regarded as the most sensitive method for the detection and evaluation of early stage osteonecrosis [36, 37]. Homogeneous intermediate or high signal intensity in T1-weight images and low signal intensity in T2-weight images were observed. However, osteonecrosis, which damages normal bone morphology, induces edema and inflammation, and increases bone marrow pressure, manifests focal inhomogeneous high signal intensity areas in T1W images, and mixed low signal-intensity area and high signal intensity areas in T2W images[23]. Our data showed homogeneous high signal intensity areas in T1W images and lower intensity signal in T2-weight images, thus demonstrating the positive efficacy of PTH on osteonecrosis.

Impaired and delayed bone healing were observed in a SAON model with CD, exhibiting osteonecrosis foci, empty lacunae, poor mechanical properties, as well as less trabecular bone, osteoblasts, reduction of osteogenesis and mineralization [10]. However, the exact mechanisms of SAON are still unclear. Abnormalities of MSCs, osteocytes and osteoblast homeostasis are generally accepted as one of the common pathways resulting in osteonecrosis [38]. Moreover, steroids could induce adipogenic differentiation of MSCs and inhibit their osteogenic differentiation [39]. Prolonged treatment with steroids could simultaneously lead to the apoptosis of osteoblasts and osteocytes [40]. PTH [1–34] exerts anabolic effects on bone and enhances bone strength by inducing new bone formation at inactive bone surfaces and by further stimulating mineral apposition. Micro-CT evaluation showed that intermittent administration of PTH[1–34] could enhance the osteogenic effect, resulting in improvement of biomechanical strength. Histological analysis revealed more osteoblasts, and fewer empty lacunae and osteonecrosis foci within and around the tunnel, as well as the increase of mineralization and osteogenesis in bone. This validated that PTH could enhance bone repair and decrease the occurrence of osteonecrosis, which might reduce the incidence of fracture after CD. Abundant fibrosis could be observed after steroid induction in our study, and much fibrosis still remains at 6 weeks after CD, which is consistent with other studies[10, 19]. Our study showed that intermittent treatment with PTH could inhibit fibrosis formation after steroid induction, consistent with similar results in a previous study [41, 42]. However, continuous PTH administration elicited an opposite effect on fibrosis. Previous studies found that PTH could increase activity, prolong the lifespan, decrease apoptosis of osteoblasts and reduce fibrosis, as well as enhance the osteogenic differentiation of MSCs [43, 44], thus providing mechanistic explanations for our findings.

Another final pathway leading to SAON is the interruption of microcirculation [45], in which steroids have been found to decrease VEGF expression and impede the process of revascularization [46]. Another study confirmed the correlation between SAON and regional endothelial dysfunction [47]. PTH has been proven to be a potent stimulator of VEGF in vitro [48], and acts as a mitogen for endothelial cells and promoter of angiogenesis. Additionally, PTH might improve neovascularization by exerting an anti-apoptotic effect. CD could also promote revascularization by increasing capillary blood flow in necrotic bone. Our findings that PTH[1–34] could partially restore vessel histomorphology to normal, might be due to the synergistic effects of CD with PTH treatment, resulting in decreased marrow pressure and improved revascularization. This can also explain our observation of increased number of vessels with PTH treatment in the tunnel, as compared to the SAON-CD group. Larger diameter of vessels in the marrow was observed, but unexpectedly, we found no significant difference in the number of vessels between the SAON-CD group and the SAON-CD-PTH group in the marrow, even though PTH could increase the proliferation of endothelial cells. PTH can inhibit adipocyte differentiation [49] and thus decrease the diameter of fat cells, as well as enhance angiogenesis, and that might possibly explain the effect on enlarging vessel diameter in the marrow.

BMP-2, bFGF, IGF-1, VEGF and RUNX2 are reported to be the downstream genes affected by PTH administration during osteogenesis and angiogenesis [29–31]. BMP-2 is regarded as an upstream osteogenic gene that modulates the promoter region of RUNX2, which is a key transcription factor associated with osteoblast differentiation. It has been demonstrated to play a crucial role in enhancing bone formation [29] by PTH through inducing osteogenesis of MSCs and increasing osteoblast activity [44]. The local MSC activity and efficacy of bone repair are correlated positively with BMP-2 expression [50]. The reduction of BMP-2 and RUNX2 expression caused by steroid in the distal femur were rescued by administration of PTH[1–34]. IGF-1 is required for PTH-stimulated bone formation [51]. We found that IGF-1 expression displayed an increasing trend after steroid induction, which is similar to the previous report [52]. The elevated expression of IGF-1 might be caused by anoxia induced by SAON. Meanwhile, PTH administration enhanced the elevation of IGF-1 expression in vivo. Basic FGF exerts effects on osteogenesis and angiogenesis [31, 53], and PTH treatment can enhance the expression of bFGF in osteoblasts [31]. The knockdown of bFGF blocks the contribution of intermittent PTH stimulation on bone formation [54]. Our results demonstrated that the expression of bFGF was impaired by steroid, which is consistent with the result of Li et al. [55]. However, these adverse effects were inhibited by the administration of PTH. Osteocalcin, which is considered a specific marker of bone formation, is thought to be a more sensitive marker than serum alkaline phosphatases level when corticosteroid is administered [56]. Low serum osteocalcin levels were found in glucocorticoid-treated patients [57]. In our study, serum OCN level was found to be markedly elevated in SAON-CD-PTH animals, as compared to the SAON-CD group, which correlates with a better capacity for bone repair. VEGF is also regarded as an essential factor in both normal and abnormal conditions of neovascularization [32, 58]. There is evidence to show that steroids could suppress VEGF production in vivo [59]. Our findings showed that both local expression and serum VEGF level was increased in the SAON-CD-PTH group, as compared with the SAON-CD group, which are consistent with the histology results.

## Conclusion

This study revealed that intermittent PTH[1–34] could promote bone repair after CD in a rabbit model of SAON, as well as enhance vascularization, as evidenced by increased number of blood vessels within the surgically constructed CD bone tunnel, and enlarged diameter of vessels outside the tunnel. These findings might thus provide a potential therapeutic strategy for patients with early-stage SAON.

## Supporting information

S1 Fig The diagram and the photo are shown. The diagram and photo of mechanical testing is displayed, with the yellow arrow denoting the orientation of the compressive force.
(TIF)

S2 Fig PTH increases the number of osteoblasts in both the tunnel and the bone marrow of SAON-CD rabbits. The quantification of osteoblasts is shown. Data are presented as mean ± SD, and error bars in the figure denote SD, *p<0.05, **p<0.01.
(TIF)

S3 Fig PTH elevates the serum calcium level. The serum calcium level was measured. Data are presented as mean ± SD, and error bars in the figure denote SD, *p<0.05.
(TIF)
