# Supplementary figures and images for: PTH[1-34] improves the effects of core decompression in early-stage steroid-associated osteonecrosis model by enhancing bone repair and revascularization
Source: PLoS One. 2017 May 31;12(5):e0178781. doi: 10.1371/journal.pone.0178781 (PMC5451136; doi:10.1371/journal.pone.0178781)

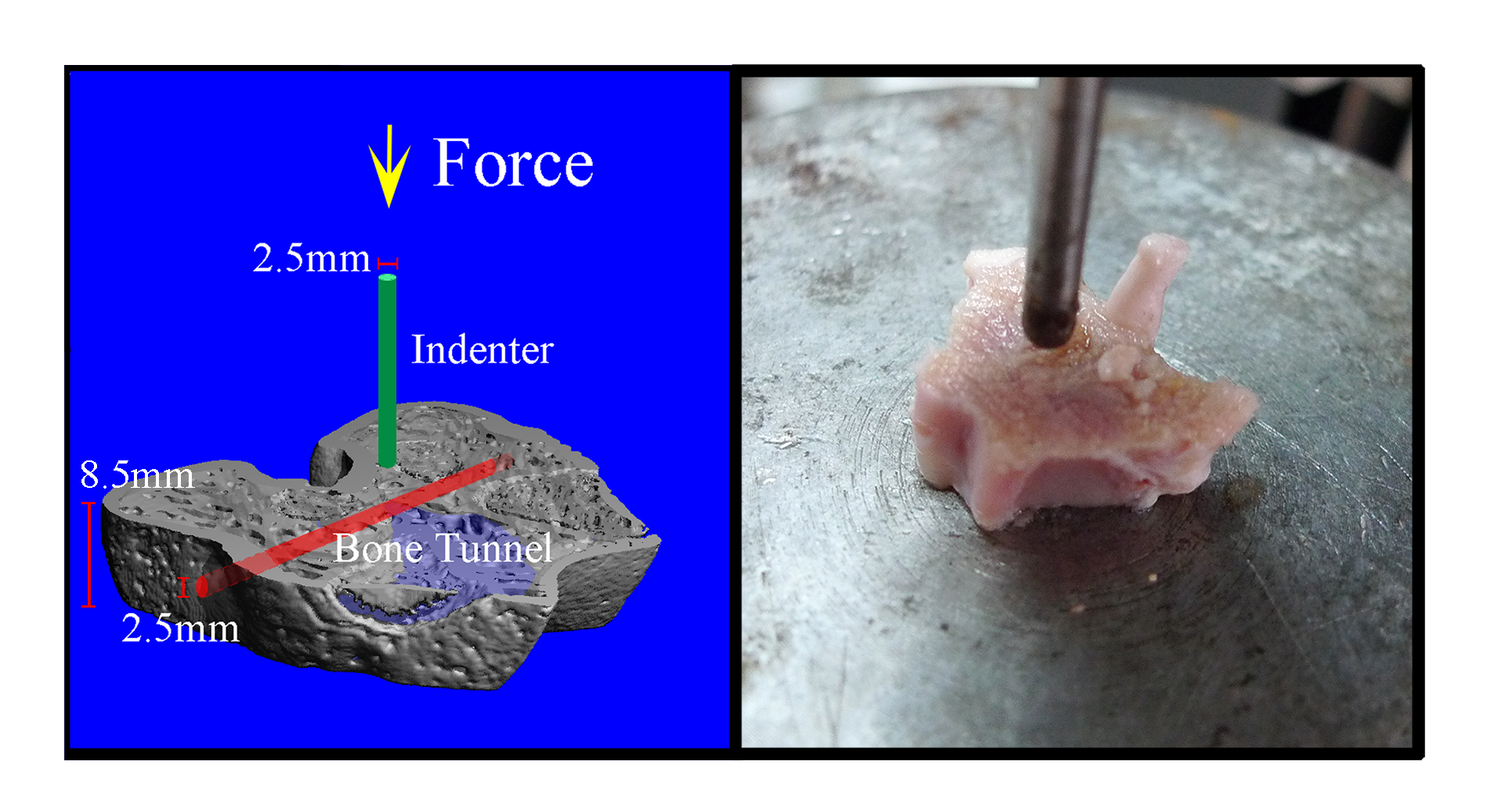

Supplement: S1 Fig — The diagram and photo of mechanical testing is displayed, with the yellow arrow denoting the orientation of the compressive force. (TIF) [file pone.0178781.s001.tif]

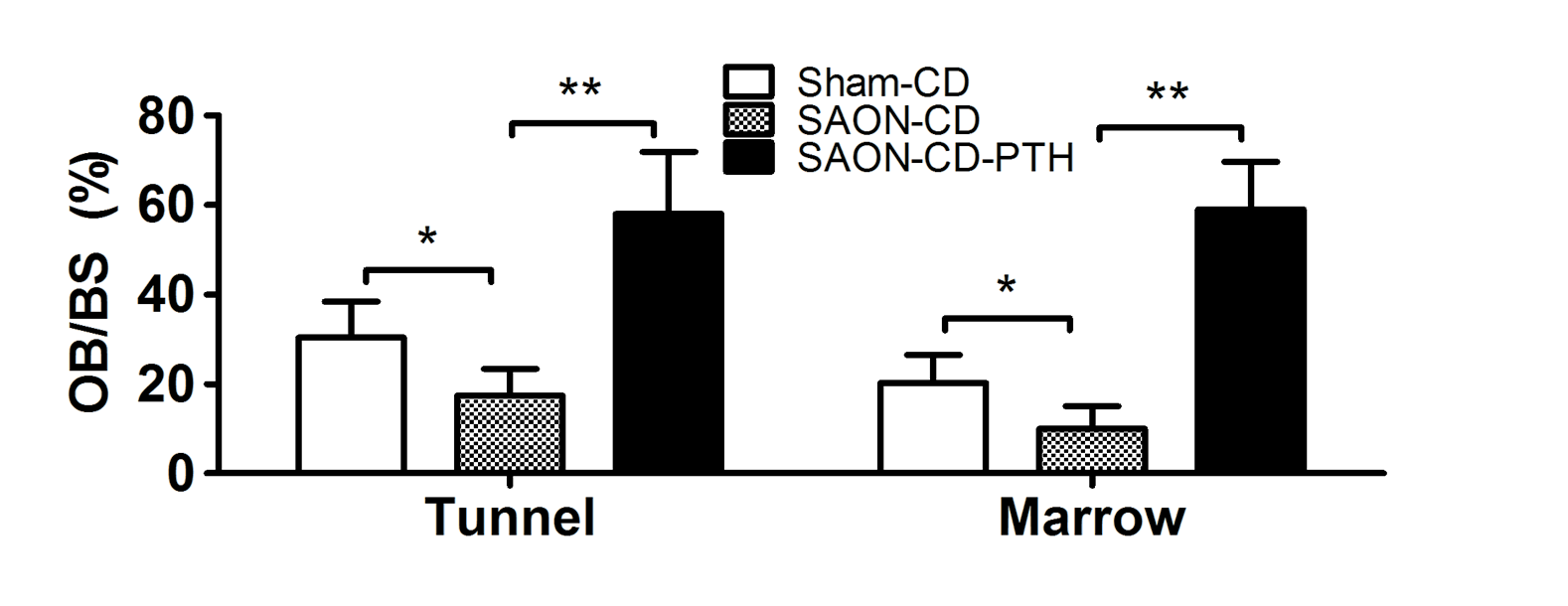

Supplement: S2 Fig — The quantification of osteoblasts is shown. Data are presented as mean ± SD, and error bars in the figure denote SD, *p<0.05, **p<0.01. (TIF) [file pone.0178781.s002.tif]

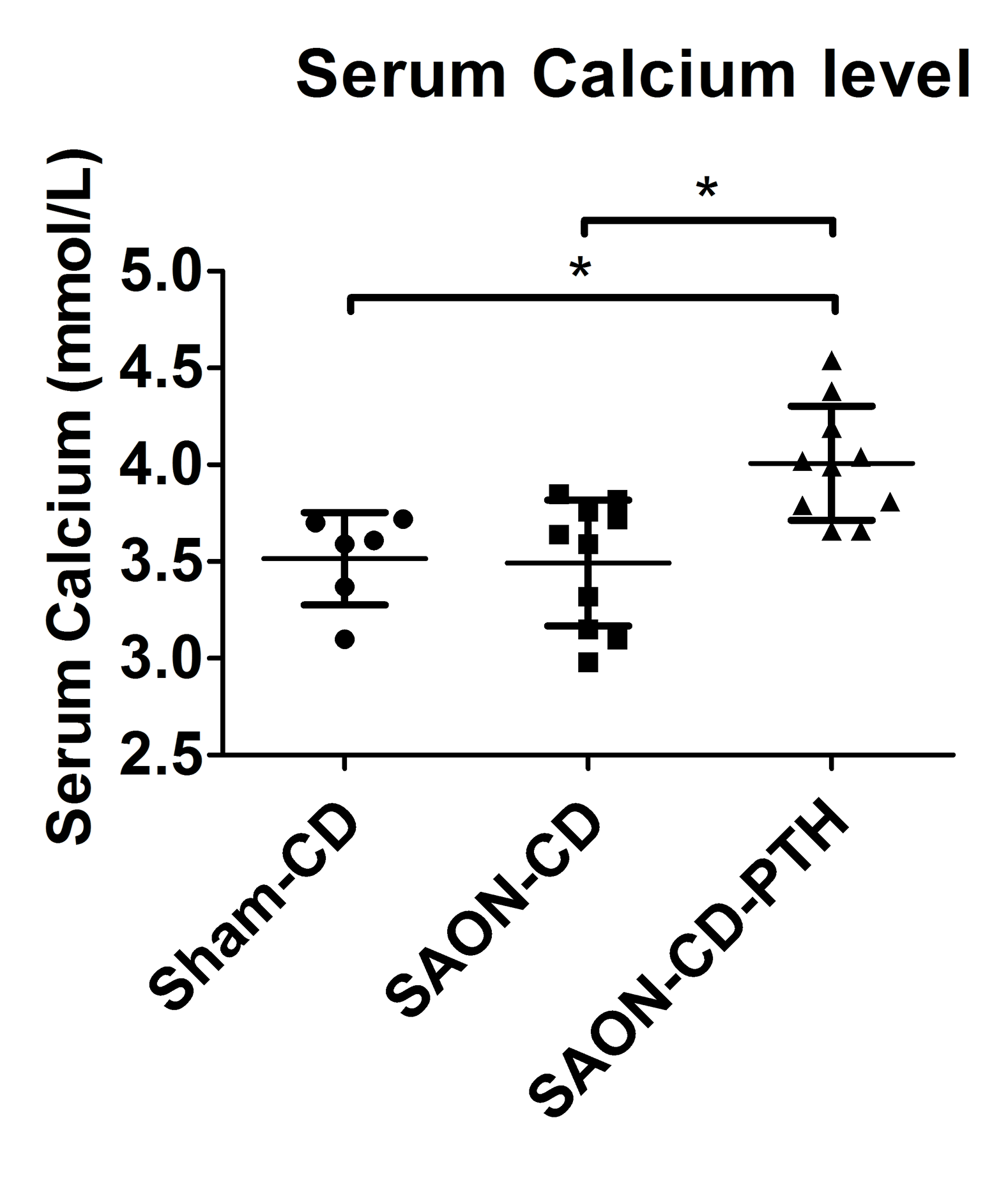

Supplement: S3 Fig — The serum calcium level was measured. Data are presented as mean ± SD, and error bars in the figure denote SD, *p<0.05. (TIF) [file pone.0178781.s003.tif]
